# Supplementary material for: The distinct roles of genome, methylation, transcription, and translation on protein expression in Arabidopsis thaliana resolve the Central Dogma’s information flow
Source: Genome Biol. 2025 Sep 29;26:319. doi: 10.1186/s13059-025-03741-0 (PMC12477803; doi:10.1186/s13059-025-03741-0)
Supplement: Supplementary file 1 — Additional file 1: All supplemental figures and legends. [file 13059_2025_3741_MOESM1_ESM.docx]

**Supplemental Figures and Legends for the paper “The distinct roles of genome, methylation, transcription, and translation on protein expression in Arabidopsis thaliana resolve the Central Dogma’s information flow” by Zhong et al Genome Biology 2025**

***Fig S1*** *Omni-C contact map as generated by PretextSnapshot for Col-0*

*
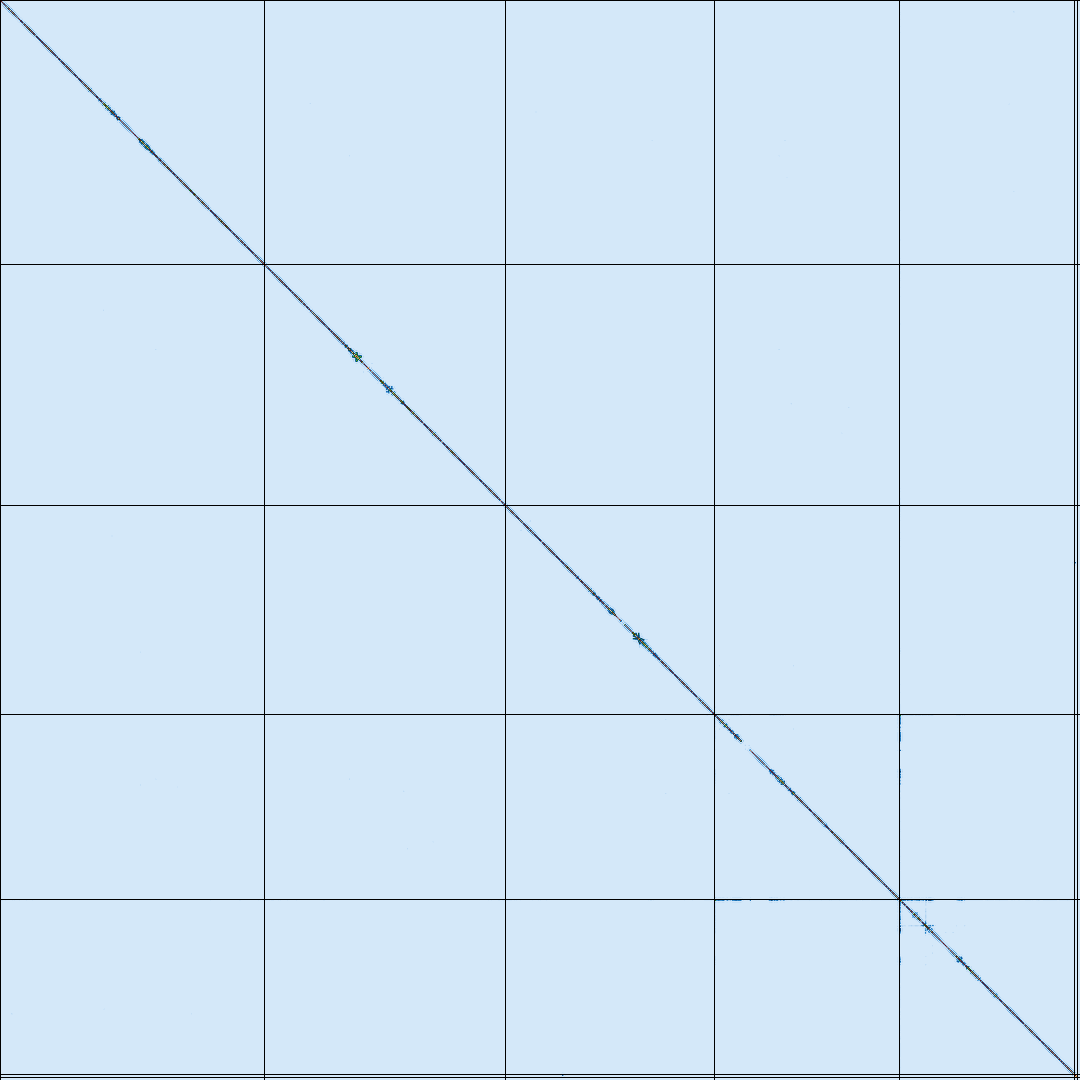
*

***Fig S2*** *Omni-C contact map as generated by PretextSnapshot for Can-0*

***
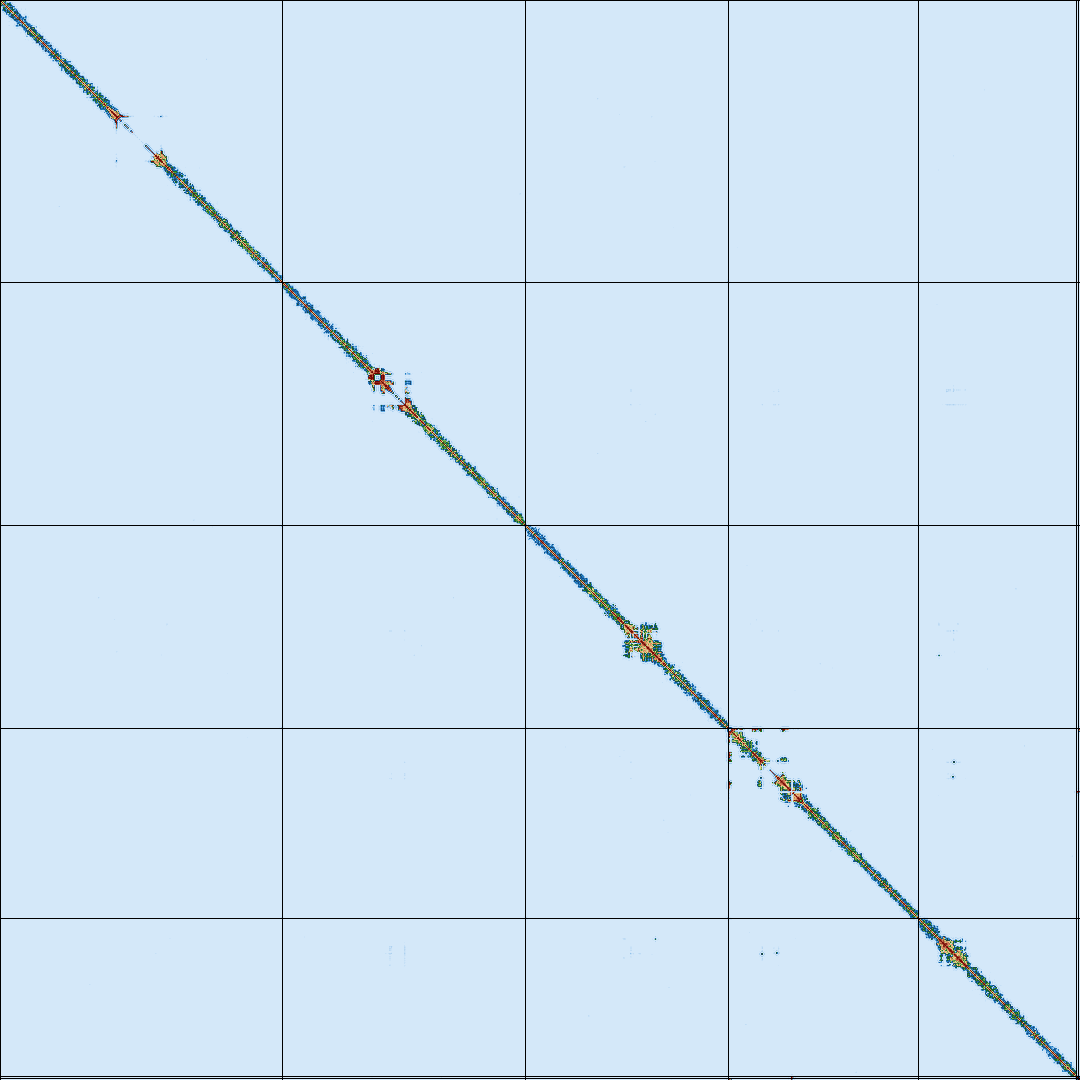
***

***Fig S3*** *Distribution of mRNA and protein expression restricted to genes expressed in all replicates, scaled so that the median level of expression of genes with both protein and mRNA expression is equal to 1. A, B: scatter plots of mRNA (x-axis) vs protein (y-axis) expression for orthologous genes in Col-0 (A) and Can-0 (B). Dotted red lines show medians. C,D: Histograms of mRNA expression for genes with (pale red) or without (grey) detectable protein expression in Col-0 (C) and Can-0 (D). The black curves indicate lognormal densities fitted to the mRNA+protein histograms using robust estimates of mean and standard deviation. Expression scales are logarithmic throughout.*

***
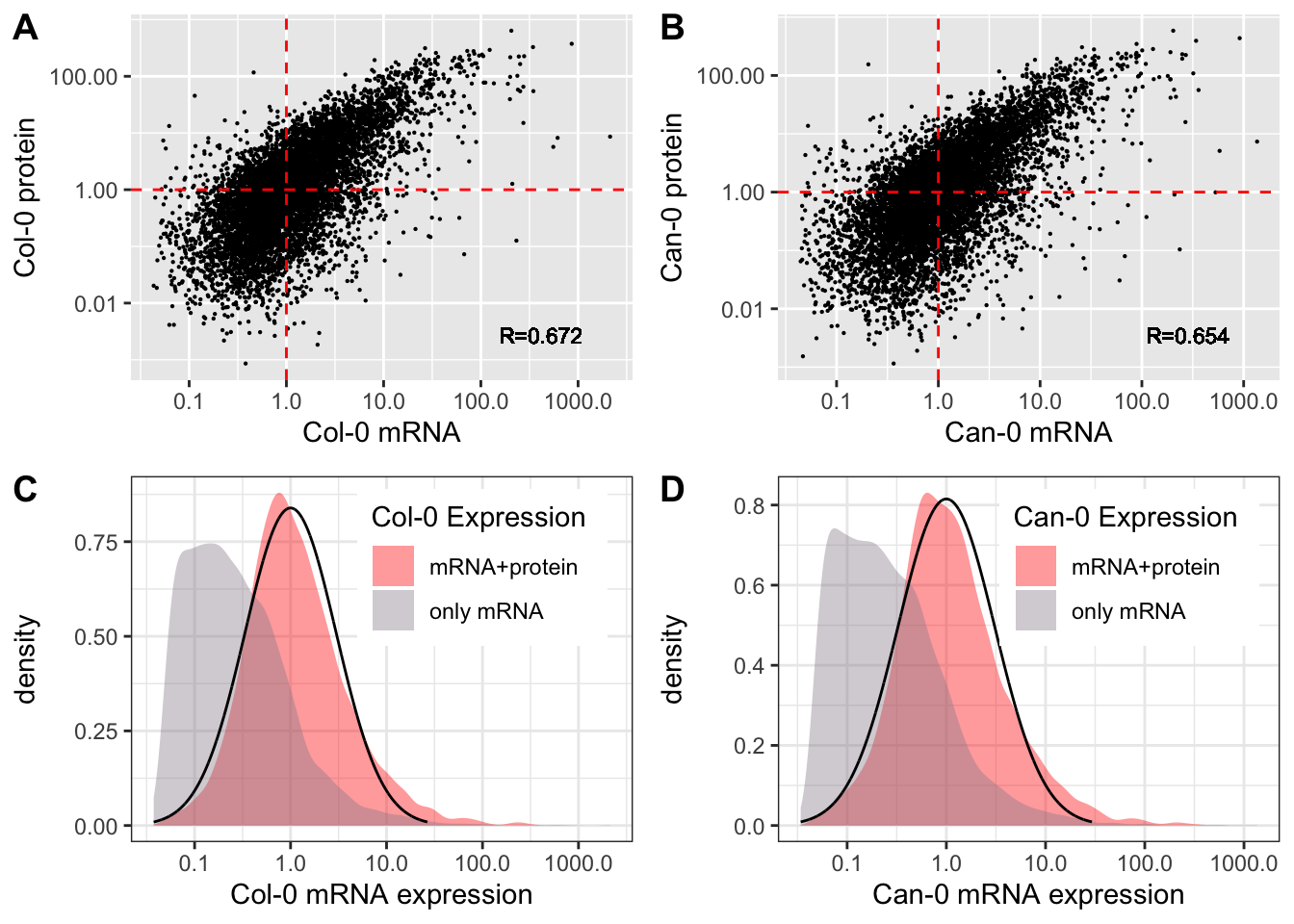
***

***Fig S4*** *Forest plot analysis of the effects of isoform count on mRNA-protein correlation. The rows represent subsets of genes annotated to have the indicated number of annotated isoforms (“# isoforms”) in Col-0 or Can-0; blue squares indicate the Pearson correlations and the red lines their 95% confidence intervals. The numbers of genes in each subset are given in the column “# genes”. The vertical blue line is the overall mean correlation.*

*
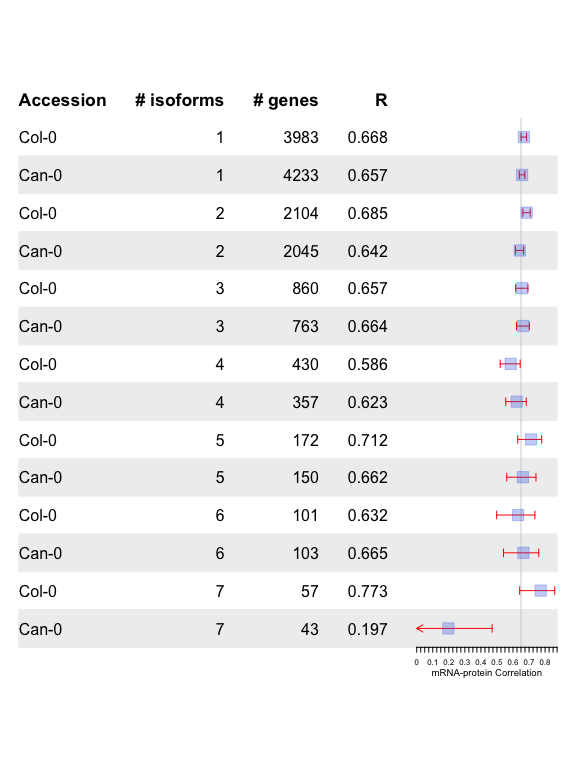
*

***Fig S5*** *Heatmaps of CpG methylation correlation between methylation estimated from bisulphite-converted Illumina vs Oxford Nanopore sequence. (A) 2.6M CpG sites in Col-0 (B) 2.8M CpG sites in Can-0.*

******

***Fig S6*** *Spatial gene density distributions for Col-0 genes without mRNA or protein expression, categorised according to gbM<70% (red) or gbM>70% (blue).*

**

***Fig S7*** *Estimated codon effects on mRNA and protein expression in Col-0. Shown are barplots representing the multiple regression coefficients for 61 non-termination codons (y-axis) for modelling log-transformed mRNA (red) or protein expression (blue). Error bars show standard errors.*

*
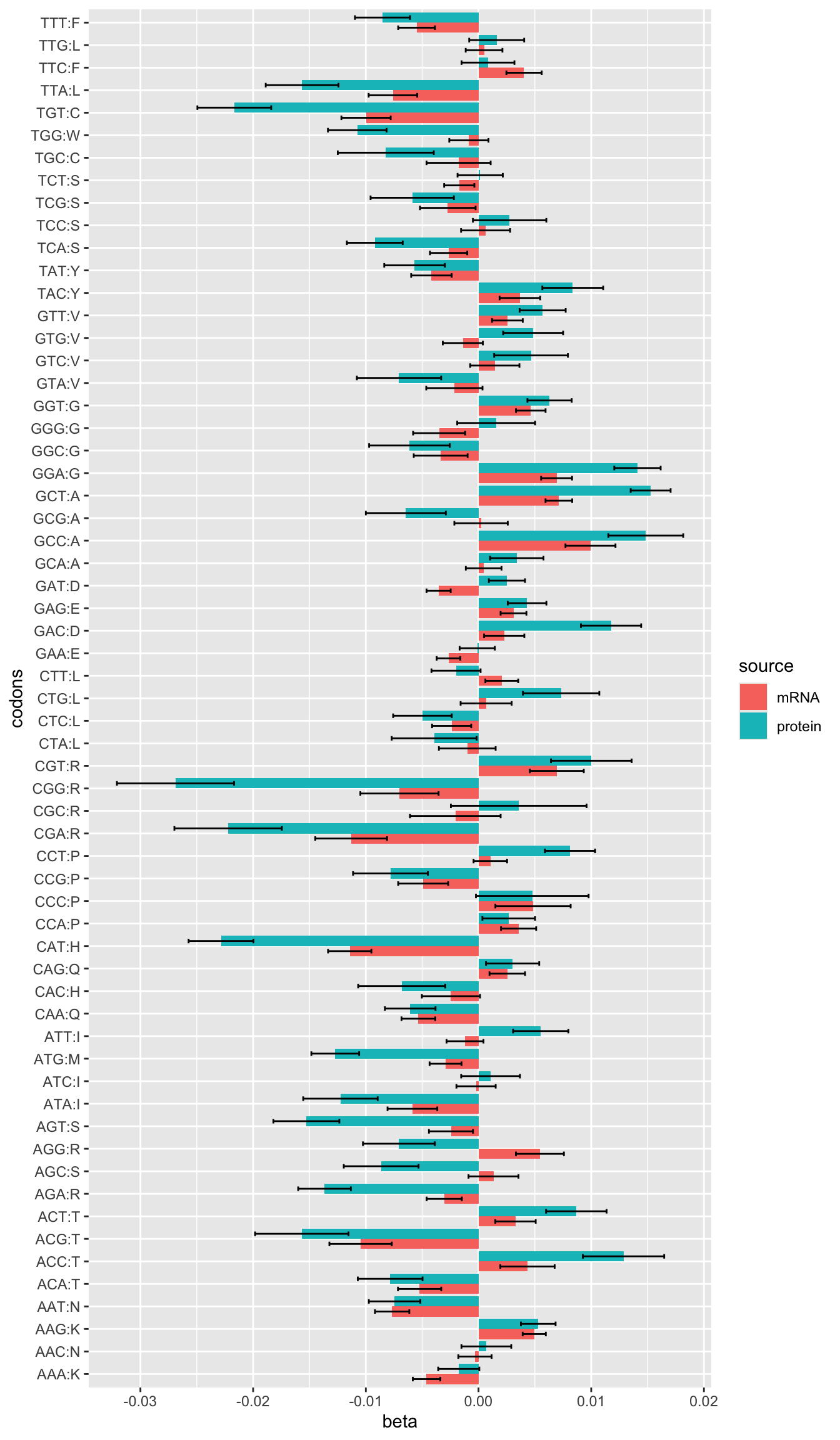
*

***Fig S8*** *Scatter plots of the estimated codon expression effects for mRNA (A, B) or protein (C, D) vs tRNA abundance in Col-0 (A, C) and Can-0 (B, D). Each dot represents one codon, labelled by its codon and encoded amino acid. The black numbers are the Pearson correlation coefficients.*

*
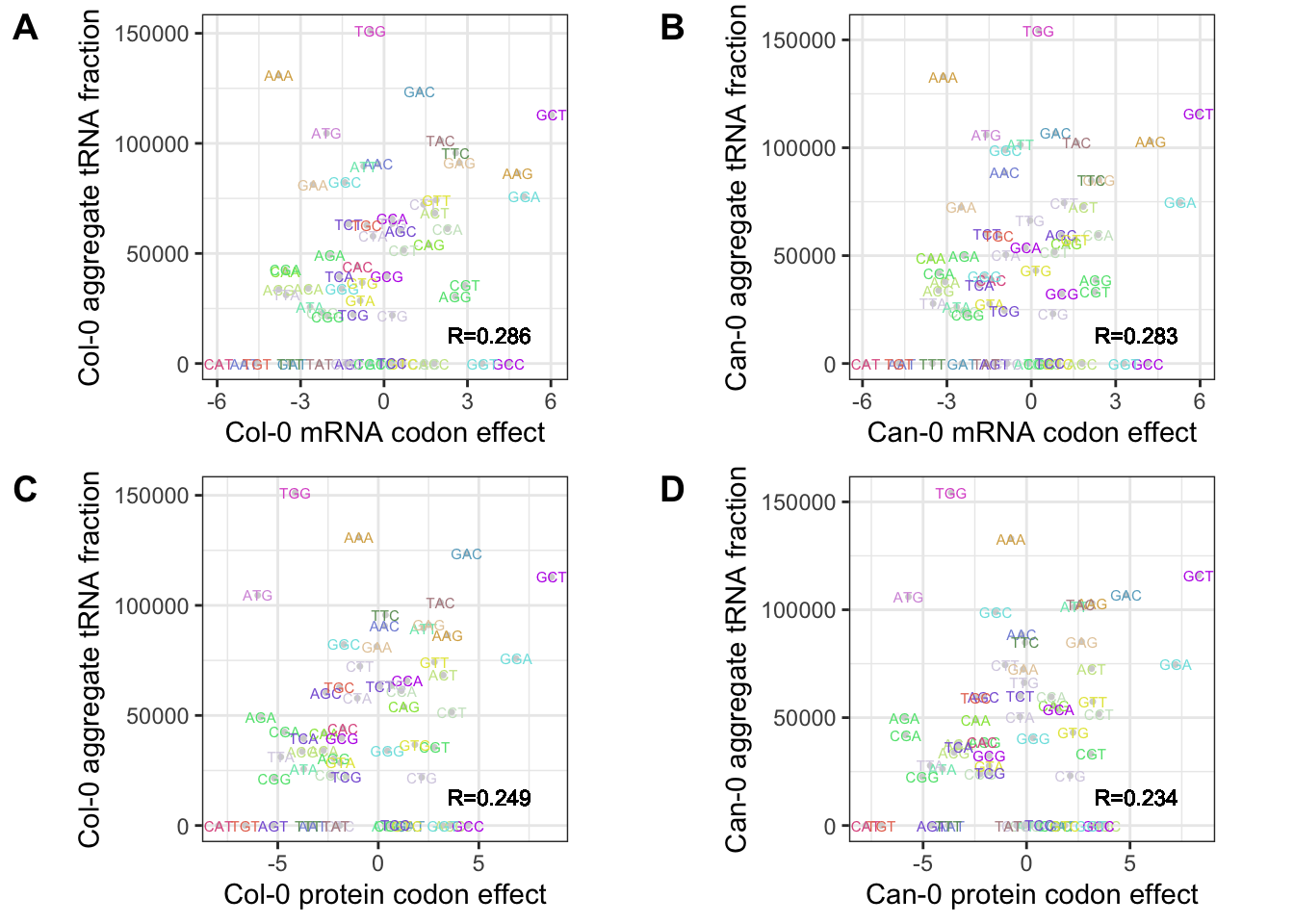
*

***Fig S9*** *Forest plot analysis of the effects on mRNA-protein correlation of codon differences between Col-0 and Can-0. The rows represent subsets of genes annotated to have the indicated number of different codons (Δ codons) between Col-0 and Can-0; squares indicate the Pearson correlations (blue: mRNA, red: protein) and the red lines their 95% confidence intervals. The numbers of genes in each subset are given in the column “# genes”.*

***
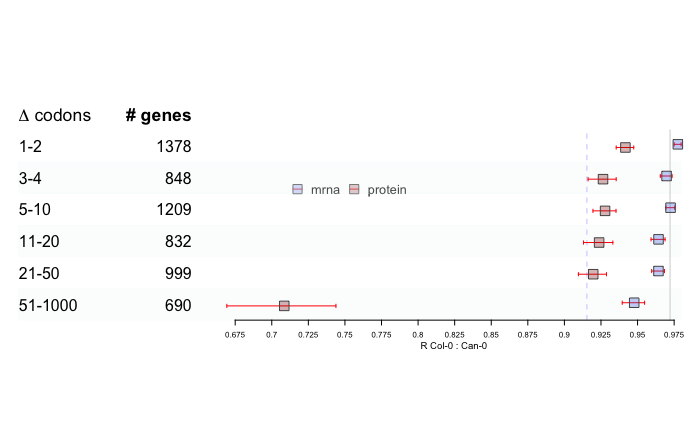
***

***Fig S10.*** *Differentially expressed mRNA transcripts. Of 17,771 transcripts quantified, 7,585 were differentially expressed (FDR <0.05). A. Volcano plot showing the relationship between statistical significance (adjusted p-value) on the y-axis and the biological significance (log2 fold change) on the x-axis. B. Gene ontology terms enrichment for different groups of differentially expressed genes (DEGs).*

******

***Fig S11.*** *Differentially expressed proteins. Of 8,915 proteins quantified, 1196 were differentially expressed (>2-fold-change, adj. p <0.05). A. Volcano plot showing the relationship between statistical significance (adjusted p-value) on the y-axis and the biological significance (log2 fold change) on the x-axis. B. Gene ontology terms enrichment for different groups of differentially expressed proteins (DEPs).*

**
