# Supplementary material for: The distinct roles of genome, methylation, transcription, and translation on protein expression in Arabidopsis thaliana resolve the Central Dogma’s information flow
Source: Genome Biol. 2025 Sep 29;26:319. doi: 10.1186/s13059-025-03741-0 (PMC12477803; doi:10.1186/s13059-025-03741-0)
Supplement: Supplementary file 2 — Additional file 2: Supplemental Tables S1 and S2 with legends. [file 13059_2025_3741_MOESM2_ESM.docx]

**Supplemental Table Legends for the paper “The distinct roles of genome, methylation, transcription, and translation on protein expression in Arabidopsis thaliana resolve the Central Dogma’s information flow” by Zhong et al Genome Biology 2025**

**Supplemental Tables S1 and S2 are included in this file. The Legends to the other Supplemental Tables are in this file but are too large to include, instead they are linked to Additional Files as indicated.**

**Additional files**

Additional file 1: All supplemental Figures and legends (MS Word, separate file).

Additional file 2: Supplemental Tables S1 and S2 with legends (MS Word, this file; all other supplemental tables are large and so are provided as separate Additional files).

Additional file 3: Table S3 Tabular text file listing Orthologous genes between Col-0, Can-0 and Araport11. Each row represents one Homologous Group (HOG). Numbers of alternatively spliced isoforms annotated for each gene are indicated by the “Transcripts” columns.

Additional file 4: Table S4 Normalised expression values for gbM, mRNA, protein and ribo-RNA. Values are supplied for each replicate (except for gbM) and combined across replicates, and log-transformed in both accessions Col-0 and Can-0 (.xlsx).

Additional file 5: Table S5 Pearson correlations between replicates for log-transformed mRNA, protein and ribo-RNA expression values. Light blue background indicates correlations between replicates within an accession, white background indicates correlations between replicates from different accessions (.xlsx).

Additional file 6: Table S6 Estimated codon effects in Col-0 and Can-0 together with the tRNA codon effects (.xlsx).

Additional file 7: Table S7 tRNA expression values for Col-0 and Can-0 (Tabular Text) .

Additional file 8: Table S8 ANOVA tables used to generate Figure 9 (.xlsx).

Additional file 9: Table S9 Differential Expression Analysis (.xlsx).

Additional file 10: Table S10 Accession numbers of sequencing data submitted to the European Nucleotide Archive (.xlsx).

Additional file 11: Analysis linking estimated codon effects to mRNA half-lives (MS Word).

**Annotations**

We have uploaded annotations of our Col-0 and Can-0 to Figshare [110]. This archive contains (i) Annotation of Col-0 (GFF3) (ii) Annotation of Can-0 (GFF3) (iii) Col-0 mRNA sequences (Fasta) (iv) Can-0 mRNA sequences (Fasta) (v) Col-0 peptide sequences (Fasta)

(vi) Can-0 peptide sequences (Fasta)

***Table S1*** *Summary statistics for our assemblies of Col-0 and Can-0, and comparisons with three published Col-0 assemblies. Base QV estimates the error rate in the assembly, expressed as the negative log10 of the probability a given base pair is erroneous* [63],  *BUSCO estimates the completeness of the gene content of the assembly in terms of 4596 single-copy orthologs found in brassicas [64, 65]. Assembly N50 is the length of contigs such that 50% of the assembly is in contigs of at least N50. Scaffold N50 is the corresponding length in assembled scaffolds. #Gaps is the number of gaps in the assembly. GC content is the percentage of G+C nucleotides in the assembly. Genome size is the total length of the assembly.*

|  |  |  |  |  |  |  |
| --- | --- | --- | --- | --- | --- | --- |
|  |  | **Col-0** | **Can-0** | **Col_CEN** | **Col_XJTU** | **Col_Tair10** |
| **Size (bp)** | **Chr1** | 32641497 | 34797179 | 32540122 | 32659241 | 30427671 |
|  | **Chr2** | 22816092 | 23408526 | 22217084 | 22560461 | 19698289 |
|  | **Chr3** | 25863195 | 24971814 | 25743512 | 26161332 | 23459830 |
|  | **Chr4** | 21559647 | 19452999 | 21578073 | 22250686 | 18585056 |
|  | **Chr5** | 29666167 | 30003755 | 29480885 | 30093473 | 26975502 |
| **Base QV** | **Chr1** | 58.84 | 55.65 | 61.44 | 67.78 | 48.46 |
|  | **Chr2** | 59.95 | 58.03 | 45.57 | 61.89 | 52.3 |
|  | **Chr3** | 51.17 | 60.07 | 57.52 | 66.16 | 51.27 |
|  | **Chr4** | 64.76 | 57.12 | 49.93 | 66.73 | 44.7 |
|  | **Chr5** | 52.61 | 61.06 | 51.36 | 63.95 | 48.76 |
| **BUSCO** | **Single** | 4501 (97.9%) | 4500 (97.9%) | 4503 (98.0%) | 4503 (98.0%) | 4503 (98.0%) |
|  | **Duplicate** | 59 (1.3%) | 62 (1.3%) | 59 (1.3%) | 59 (1.3%) | 58 (1.3%) |
|  | **Fragmented** | 3 (0.1%) | 3 (0.1%) | 2 (0.0%) | 2 (0.0%) | 3 (0.1%) |
|  | **Missing** | 33 (0.7%) | 31 (0.7%) | 32 (0.7%) | 32 (0.7%) | 32 (0.6%) |
| **Assembly N50** | | 18,399,411 | 21,470,873 | - | 22,250,686 | 11,194,537 |
| **Scaffold N50** | | 25,863,195 | 24,972,360 | - | 26,161,332 | 23,459,830 |
| **#Gaps** | | 17 | 5 | - | 2 | 93 |
| **GC content** | | 36.28% | 36.31% | - | 36.34% | 36.03% |
| **Genome size** | | 133,233,343 | 133,091,828 | 131,559,676 | 133,725,193 | 119,668,634 |

***Table S2*** *Counts of differences as computed by dna_diff* [20] *between our Can-0 and Col-0 assemblies and with four other Col-0 and one other Can-0 assemblies, namely Can-Lian, Col-Lian: [16], Col-CEN* [17] *, Col-XJTU* [18] *, Col-TAIR, the TAIR10 reference, Col-CC: the community consensus assembly (Genbank id GCA_028009825.2). Data are plotted in Figure 2.*

*(a) Comparisons between our Col-0 assembly and other published Col-0 assemblies*

|  | | | |  |  |
| --- | --- | --- | --- | --- | --- |
| ***Measure*** | ***ColvsCol-CC*** | ***ColvsCol_CEN*** | ***ColvsTAIR10*** | ***ColvsCol_XJTU*** | ***ColvsCol_Lian*** |
| ***UnalignedBases_Mbp*** | *1.11917* | *0.126144* | *0.267946* | *0.112954* | *1.559004* |
| ***Breakpoints*** | *2015* | *619* | *1552* | *837* | *6131* |
| ***Relocations*** | *46* | *19* | *77* | *11* | *44* |
| ***Translocations*** | *2* | *0* | *6* | *2* | *4* |
| ***Inversions*** | *3* | *2* | *28* | *0* | *2* |
| ***Large_Insertions*** | *1137* | *182* | *266* | *361* | *3978* |
| ***Tandem_duplication_insertion*** | *5* | *56* | *55* | *32* | *29* |
| ***Single_SNPs*** | *9101* | *9895* | *9345* | *2353* | *28062* |
| ***Single_Nucleotide_Indels*** | *17561* | *11006* | *6894* | *4674* | *24916* |

*(b) Comparisons between selected other Col-0 assemblies*

|  | | |  |  |
| --- | --- | --- | --- | --- |
|  | ***TAIR10vsCol_CC*** | ***TAIR10vsCol_CEN*** | ***TAIR10vsCol-XJTU*** | ***Col-CCvsCol-CEN*** |
| ***UnalignedBases_Mbp*** | *0.715504* | *0.12471* | *0.124184* | *0.140906* |
| ***Breakpoints*** | *18078* | *12949* | *14007* | *724* |
| ***Relocations*** | *61* | *64* | *63* | *24* |
| ***Translocations*** | *3* | *1* | *4* | *6* |
| ***Inversions*** | *30* | *26* | *30* | *1* |
| ***Large_Insertions*** | *9857* | *6569* | *7076* | *198* |
| ***Tandem_duplication_insertion*** | *37* | *51* | *40* | *32* |
| ***Single_SNPs*** | *10213* | *11844* | *10791* | *10545* |
| ***Single_Nucleotide_Indels*** | *4670* | *8076* | *5600* | *9173* |

*(c) Comparisons between our Can-0 assembly and other assemblies*

|  | | |
| --- | --- | --- |
|  | *ColvsCan* | *CanvsCan_Lian* |
| *UnalignedBases_Mbp* | *26.473666* | *2.894566* |
| *Breakpoints* | *14507* | *7603* |
| *Relocations* | *205* | *52* |
| *Translocations* | *344* | *24* |
| *Inversions* | *119* | *0* |
| *Large_Insertions* | *5567* | *5674* |
| *Tandem_duplication_insertion* | *63* | *51* |
| *Single_SNPs* | *682351* | *22036* |
| *Single_Nucleotide_Indels* | *561365* | *14321* |

***Table S3*** *Orthologous genes between Col-0, Can-0 and Araport11. Each row represents one Homologous Group (HOG). Numbers of alternatively spliced isoforms annotated for each gene are indicated by the “Transcripts” columns.* ***Data are in Additional File 3***

***Table S4*** *Normalised expression values for gbM, mRNA, protein and ribo-RNA. Values are supplied for each replicate (except for gbM) and combined across replicates, and log-transformed in both accessions Col-0 and Can-0.* ***Data are in Additional File 4***

***Table S5*** *Pearson correlations between replicates for log-transformed mRNA, protein and ribo-RNA expression values. Light blue background indicates correlations between replicates within an accession, white background indicates correlations between replicates from different accessions****. Data are in Additional File 5***

***Table S6*** *Estimated codon effects in Col-0 and Can-0 together with the tRNA codon effects.* ***Data are in Additional File 6***

***Table S7*** *tRNA expression values for Col-0 and Can-0.* ***Data are in Additional File 7***

***Supplemental Table S8*** *ANOVA tables used to generate Figure 9. Summary of omics multiple regression models, relating to the barplots the Figure. Each block of consecutive rows with the same background shade share the same Model specification ^a^. Each row describes the variance component listed as the source of variation ^b^ . The degrees of freedom for the component in the analyses of variance is in column df ^c^. The negative base-10 logarithm of the p-value for the variance component is in column logP ^d^. The percentage of variance explained by the component after fitting the preceding components in the model is in column R^2^%^e^ (these are the values displayed in the stacked barplots in Figure 9). The cumulative percentage of variance explained by the components is in column Cum R^2^ %****^f^****. Each model is in the format Y ~ X, where Y is the dependent variable and X is one or more independent explanatory variables. The dependent variables for the Col-0 analyses are:- Col-protein, log-transformed protein expression; Col-mRNA: log-transformed mRNA expression for genes also with protein expression; Col-mRNA*: log-transformed mRNA expression for genes without protein expression; Col-gbM: percent gene-body methylation for genes also with protein expression; Col-gbM*: percent gene-body methylation for genes without protein expression. Similar names apply for the Can-0 analyses. The independent explanatory variables are:- CDS: DNA sequence composition; gbM: percent gene body methylation; mRNA: log-transformed mRNA expression. CDS effects are subdivided into protein.length, amino.acid.usage and codon.usage;* ***Data are in Additional File 8***

***Supplemental Table S9*** *Differential Expression analysis between Col-0 and Can-0. Workbook DE mRNA shows the EdgeR analysis output for mRNA. Workbook DE protein shows the analysis output for protein.* ***Data are in Additional File 9***

***Supplemental Table S10*** *Samples and ENA accession numbers. See also the statement in “Availability of data and materials”* ***Data are in Additional File 10***
